# Co-Attendance Communities: A Multilevel Egocentric Network Analysis of American Soccer Supporters’ Groups

**DOI:** 10.3390/ijerph18147351

**Published:** 2021-07-09

**Authors:** Adam R. Cocco, Matthew Katz, Marion E. Hambrick

**Affiliations:** 1 Department of Health and Sport Sciences, University of Louisville, Louisville, KY 40292, USA; marion.hambrick@louisville.edu; 2 Department of Sport Management, University of Massachusetts, Amherst, MA 01003, USA; mkatz@isenberg.umass.edu

**Keywords:** social networks, egocentric network analysis, consumer behavior, sport fans, soccer

## Abstract

The growth of professional soccer in the United States is evident through the rapid expansion of franchises and increased game attendance within Major League Soccer (MLS) and the United Soccer League (USL). Coinciding with this growth is the emergence of European-style supporters’ groups filling sections of MLS and USL stadiums. In this study, the authors utilized an egocentric network analysis to explore relationships among supporters’ group members for two professional soccer clubs based in the United States. Egocentric network research focuses on the immediate social environment of individuals and is often viewed as an alternative approach to sociocentric (i.e., whole network) analyses. This study employed hierarchical linear modeling as an example of multilevel modeling with egocentric data, using ego- and alter-level variables to explain the strength of co-attendance ties. The results indicate the perceived commitment of fellow fans to the team, shared membership in a supporters’ group, age, and interactions with other fans in team settings related to higher levels of co-attendance. The outcomes of this study are both theoretical, as they advance an understanding of sport consumer behavior within soccer supporters’ groups, and methodological, as they illustrate the unique value of employing egocentric network analysis in sport fan research.

## 1. Introduction

The rapid ascendance of professional soccer in the United States within the last quarter of a century has changed the American sports landscape. Major League Soccer (MLS), the top-tier soccer division in the United States, averaged over 21,000 attendees per game during the 2019 season after struggling to draw 15,000 fans to games for much of its first 15 years of existence [1]. Similarly, the United Soccer League (USL), the second-tier professional soccer division in the United States, has almost doubled its average game attendance during the 2010s, with the 2019 season bringing in nearly 4500 fans per game [1]. In addition, recent polls indicated more than 50% of 18-to-34-year-olds held an active interest in the sport of soccer, putting it on par with the sport of basketball among this key age demographic and suggesting this growth of soccer fandom in the United States may be sustainable over the long term [2,3]. Alongside the growth of professional soccer in the United States has been the emergence of dedicated soccer supporters’ groups [4], a phenomenon with strong tradition among European soccer clubs but a relatively novel concept within the American sports landscape. Soccer supporters’ group members differ from traditional sport fans through their expressions of organized fandom. Commonly, these groups occupy a specific section of the stadium together and exhibit flags, banners, and other visual displays; organize cheers and chants; and play musical instruments to enhance the in-stadium atmosphere and provide a more active and engaged form of support for their team [4,5,6,7]. To orchestrate this type of collective behavior requires coordination and social interaction among group members. Therefore, soccer supporters’ groups rely upon social networks, or collections of individuals who share relationships [8], to advance goals related to their fandom. Yet, little is known about the network characteristics of these groups or how relationships among group members impact sport consumption behaviors [9]. For example, does shared membership in the same supporters’ group impact game attendance? What about variance in other attributes between fans, such as age, gender, or level of team identification? Research providing insight on these questions can help sport organizations understand factors impacting positive fan behavior, such as increased game attendance. Therefore, the purpose of this study was to explore the relationships formed and behaviors exhibited among individuals associated with soccer supporters’ groups utilizing a multilevel egocentric network analysis.

The resurgence of professional soccer fandom in the United States during the 2010s correlated with expansion at the beginning of the decade into new markets with established supporters’ groups, particularly Seattle (Emerald City Supporters) and Portland (Timbers Army) [4,10]. The success of these organizations at the ticket office, as well as the in-stadium atmosphere created by these groups, changed the way MLS viewed organized supporter culture and forced MLS to recognize the value in supporting and highlighting organic soccer fandom [10]. Since this realization, MLS has pivoted to a new marketing strategy and now promotes its most visual and vocal fans as the heart of soccer culture in the United States. The results from this new strategy have been successful. Atlanta United, for example, averaged approximately 50,000 fans per game since its inaugural season in 2017 [1] despite being located in a market with relatively little historical support for soccer. These attendance numbers have been driven largely by the passion and enthusiasm of members from four official supporters’ groups (Footie Mob, Resurgence, Terminus Legion, and Faction). Other recent entrants into MLS, including FC Cincinnati, Orlando City, and Minnesota United FC, began their existence in USL and developed an organic supporter club culture at the second-division level before joining MLS as expansion franchises. Each of these organizations ranks in the top half of MLS in terms of average game attendance [1], in large part due to the fan culture developed during their time in USL.

These examples of growing soccer support in the United States underlie a phenomenon well understood by sport fan researchers: individuals with higher levels of team identification are more likely to display higher levels of consumption behaviors. Traditionally, team identification studies have investigated the strength of a fan’s identification with the team itself [11,12]. However, recent research suggests team identification is not the only way to build strong bonds between fans and sport organizations. Fan-to-fan relationships can significantly influence sport consumer attitudes and behaviors [13,14], indicating fan communities serve as an important conduit to achieving outcomes traditionally linked with team identification. In fact, in a study of Japanese soccer fans, attachment to a fan community was the only salient predictor of future attendance [15]. Without an ability to maintain strong fan-to-fan relationships, individuals may cease game attendance regardless of their level of team identification [13]. Given that soccer supporter group members display high levels of identification with their club [16], it is important to understand how an individual’s association with a supporters’ group, and the relationships developed through a fan community, can impact their behaviors.

This study seeks to investigate the topic by positing that soccer supporters’ groups represent social networks. A fundamental assumption of the social network approach is that ties among actors in a social network matter [17] and these relationships influence behaviors [8]. A growing number of studies have applied social network analysis to sport in a variety of ways, and numerous scholars have called for increased application of social network analysis within sport [18,19]. Thus far, scholars have answered this call within an array of sport settings ranging from studies of team cohesion [20] to citation networks [21].

Many of the existing network studies in sport journals utilize a version of a bounded network—or some sort of defined boundary around the population of actors included in a network. In sociocentric networks, or “whole-network” studies, such a boundary is a fundamental requirement of examining the network. Some boundaries are clear, such as all athletic directors and senior woman administrators within the National Collegiate Athletic Association (NCAA) [22] or all members of a particular team [23]. In such cases, there is a clear boundary (e.g., institutions within the NCAA), and all members within this boundary are included in the network. Some other boundaries require a bit more exploration prior to data collection. Wäsche [24], for example, began with a list of potentially relevant sport tourism organizations in a particular geographical location and, after surveying each of the potential 45 actors, decided to include 37 of them in the network. The network boundary thus became the 37 identified organizations. Hambrick, Svensson, and Kang [25] similarly had to identify all active agencies in a sport for development coalition; those organizations deemed part of the coalition were included in the network and those who were not were excluded. Establishing a network boundary is a key consideration in sociocentric network studies, yet not all network studies require such a boundary nor benefit from one.

In some cases, establishing a network boundary is both unrealistic and potentially inappropriate given the setting. As an example, how might scholars define the population of all Manchester United fans, or the population of fans interested in the Summer Olympics? Where would scholars even begin to conceptualize such a network boundary? Social media researchers might define a network by all users adopting a particular hashtag [26] or all followers of a particular Twitter account [27]. Both represent innovative ways to conceptualize a fan network, yet both clearly capture only those fans engaging on that particular platform. One other approach to identify boundaries of sport fans involves formalized supporters’ groups, an area where a formalized boundary potentially exists. Katz et al. [9] initiated such a study, identifying the boundary of their fan population as official members of one particular supporters’ club. Using the population of official members, Katz et al. [9] examined the consumption and socializing networks of the members to model how network variables impact consumer behavior. However, how rigid are the population boundaries of a supporters’ club? By only examining actors explicitly within the group, those members indirectly connected to group members (e.g., members’ friends or members of another supporters’ group for the same team) who might impact the network of the group are left forgotten. Once a network boundary is established, those on the outside of the population are excluded from the network.

When it comes to sport fans, even those in supporters’ groups, we argue the network boundary is more fluid than rigid, where potentially both those within and outside the supporters’ group affect the actors within a network. The structure of a sport fanbase or fan community resembles the multiple in-group identity framework [28], which posits that sport consumers belong to subgroups within a superordinate identity. As such, fans can belong to multiple subgroups and interact across those groups. This definition aligns well with the reality of soccer supporters’ groups, where multiple subgroups support the same soccer team (superordinate identity for the fan). It is common for soccer clubs to recognize several official supporters’ groups (subgroups) and common for fans to identify as a member of more than one subgroup. This makes sense given multiple supporters’ groups for a soccer team will commonly mingle together during pregame tailgate parties, congregate together in the same section of a stadium during a match, and participate in the same displays of vocal and visual fan support for their team. Additionally, it is reasonable to assume supporters’ group members interact with individuals who do not identify as a member of a particular subgroup, yet still maintain strong identification with the team. Therefore, it becomes difficult—and perhaps inappropriate—to place rigid boundaries around supporters’ group networks, making sociocentric network analysis of these groups less reliable.

To alleviate the issues surrounding a sociocentric network analysis of soccer supporters’ groups, this study employed an egocentric network approach. Egocentric network analysis offers several conceptual and theoretical advantages over sociocentric networks when studying sport fans. Egocentric network studies remove the rigid boundaries established by a sociocentric network and instead place the focus on an individual’s relationships within their social environment [29]. Crossley et al. [30] suggested three main advantages of an egocentric network analysis. First, it provides an avenue for analyzing large networks where a complete mapping of actors in a network is not possible or realistic due to network size. Second, it is compatible with most techniques commonly found within social science research, ranging from purely descriptive statistics to structural equation modeling. Third, egocentric network analysis allows for intersecting and overlapping social circles. Therefore, this analysis technique better mimics real-world situations where people live their lives simultaneously within multiple social circles (e.g., family, friends, work, religious institutions). Employing a sociocentric network analysis technique could fail to properly bound this myriad of social circles, thereby missing key actors affecting one’s social network.

**Figure 1:**
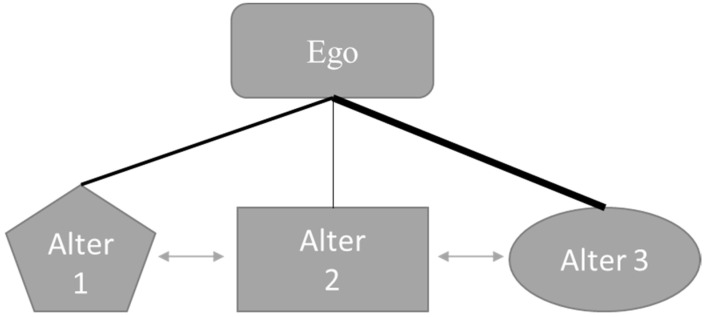
Example of an egocentric network with multilevel modeling.

The visual presented in Figure 1 demonstrates a generic egocentric network. In this fictional example, a main actor (ego, e.g., a fan of a soccer team) shares a relationship with three individuals (alters, e.g., other fans that attend matches with the ego). Each alter is represented by a different shape in the diagram because each alter possesses a unique set of attributes (e.g., age, gender, level of team identification). Additionally, the line connecting the ego to each alter varies in weight, indicating varying frequencies of interaction between the ego and their alters. Finally, lines connect Alter 1 to Alter 2 and Alter 2 to Alter 3, indicating the alters within an ego’s network can also share relationships among each other.

Sport scholars have used egocentric network approaches, either as case studies of a particular network or through larger data collections and more traditional statistical modeling. To illustrate the difference, Naraine and Parent [27] examined central users in two different national sport organization egocentric networks, treating each egocentric network like a sociocentric network. This is a reasonable and appropriate technique for examining egocentric networks. However, egocentric research can also be used to study random samples of egocentric networks and examine the results through standard statistical analysis. Katz et al. [14] used structural equation modeling to explain attendance behaviors with egocentric network variables among college hockey fans, and Katz, Heere, and Melton [31] used egocentric network analysis to predict season ticket holder retention. Using random samples, incorporating standard social science statistical modeling, and removing the need for bounded populations reflect the advantages of egocentric research as proposed by Crossley et al. [30]. Additionally, when scholars are interested in outcome variables at the ego level of analysis, standard regression modeling is appropriate [29].

Yet, when scholars condense, or amalgamate, information about specific alters into a general statistic for each ego, they inherently lose richness in the data. Such aggregation ignores the variance among alters. With whom the ego interacts matters; the individual attributes, behaviors, and attitudes of alters undoubtedly influence the ego. Variances exist among such alter characteristics because not every alter with whom an ego interacts is identical. To incorporate the variance among alters while controlling for dependence on the same ego requires a slightly different approach than the aforementioned egocentric studies: multilevel modeling [29]. Similar to commonly used hierarchical techniques, such as students nested in classrooms or employees nested in organizations, multilevel networks account for the dependence of both the intercept and slope [32]. In egocentric multilevel modeling, alters are nested in an ego as a way to control and utilize the dependence associated with such hierarchical data structures [33]. Figure 1 demonstrates multilevel modeling within a generic egocentric network. Theoretically, such a structure assumes both alter- and ego-level variables affect the outcome of interest [29,30]. One recent study of National Football League (NFL) fans used egocentric multilevel modeling to explain how co-consumption among fans generates emotional support [34]. The authors found that alter-level attributes (examined as the relationship of an alter to an ego, e.g., the alter is a friend or the alter is a family member), ego-level attributes (e.g., size of egocentric networks), and ego–alter ties (e.g., outside communication) each explained the variance in the dependent variable.

Returning to the current study of soccer supporters’ groups, we operationalized co-attendance as the primary variable of interest. In this research context, co-attendance is used to describe communal or collaborative attendance where fans attend games with other fans. United States-based soccer clubs have reported sizeable increases in attendance and have attributed much of this growth to the inclusion and promotion of soccer supporters’ groups [10]. Thus, it becomes important to explore factors that could influence attendance, particularly attendance with other fans. Based on our review of the sport marketing literature, we hypothesize that ego attributes, alter attributes, and other ego–alter ties could each affect the strength of co-attendance. In terms of ego attributes, we expect standard consumer behavior attributes such as team identification [11], hours spent on team-related social media [35], number of years as a fan [9], and whether the ego is an official member of a supporters’ club [9,13] to all significantly affect co-attendance. We also expect network characteristics to affect the co-attendance strength, such as the network size, density, and heterogeneity, consistent with previous studies on fan egocentric networks [31,34]. Yet, we also expect alter attributes to impact co-attendance ties. We hypothesize that when an ego perceives their alter to be a highly committed fan, co-attendance ties are stronger than for alters of whom the ego does not perceive to be a highly committed fan [34]. Other alter attributes, such as the alter being a member of the same supporters’ group as the ego [9] or the relational classification (e.g., family, friend), may also affect the strength of co-attendance. Finally, we hypothesize that other ties between the ego and alter may impact the strength of co-attendance ties, including how often the ego and alter communicate via social media and how often they socialize in ways not related to the focal soccer team.

For this Special Issue on the role of social networks in sport, we emphasize the value of examining sport fans via an egocentric multilevel modeling approach. Such a methodological approach allows for including boundary spanners, non-group members, and other overlapping social ties in a network analysis. Additionally, conceptualizing egocentric networks as multilevel structures allows for the exploration of both ego- and alter-level variables. Supporters’ club members do not only belong to their particular group; they are members of the larger superordinate group (i.e., the soccer club; [28]) and presumably interact with members outside their own group. Through an egocentric approach, we examine the relationships formed between fans and other supporters of the same team and employ multilevel modeling to examine how characteristics of the focal fan (i.e., ego), characteristics of fans with whom the ego interacts (i.e., alters), and the resulting network structure affect co-attendance behavior.

## 2. Materials and Methods

### 2.1. Sample and Data Collection

This research collected data about individuals associated with soccer supporters’ groups for one MLS team and one USL team located in the Midwest region of the United States. The MLS club features five team-recognized supporters’ groups, while the USL club features three official soccer supporters’ groups. A survey hosted on Qualtrics was distributed online through social media sites centered around interaction among supporters’ group members related to the teams included in this research. Although these social media sites focus on news and events related to a supporters’ group, participation is not restricted to official members of the supporters’ group. Therefore, both official supporters’ group members and non-members who associate with a supporters’ group were included in the data collection. Including non-members in this analysis supports the need for an egocentric approach. A traditional sociocentric network approach bounded around the population of official supporters’ group members would prevent the possibility of examining the impact of interactions between group members and non-group members on attendance behavior.

To meet inclusion criteria for this study, participants needed to demonstrate a willingness to answer questions related to their participation in a soccer supporters’ group. They needed to agree with an internal review board (IRB) notification statement, self-identify as a fan of one of the two teams included in this study, indicate their membership status in one of the recognized supporters’ groups for their team, and be 18 years of age or older. We secured permission from social media site administrators prior to posting an announcement and survey link on any soccer supporters’ group social media page.

### 2.2. Instrumentation

The instrument utilized to collect data for this research contained seven distinct sections. The first section (alters) was based on standard egocentric network methodology, utilizing the name generator approach [29,30]. The first step in a name generator survey asks participants (i.e., ego) to elicit members of their networks. More specifically, we asked participants to list all the individuals with whom they attended games of their preferred team during the current or previous season. Participants were instructed to list anyone they either met at the stadium, traveled to the stadium with, intentionally sat beside, or interacted with before or after the match. Each ego could identify a maximum of ten alters. This information provided the basis for creating egocentric networks for each respondent.

The second section of the survey instrument (ego–alter ties) gathered additional information about each ego–alter tie. More specifically, for each name listed in the name generator, participants were asked to indicate the number of games they attended with each alter during the previous season. Additionally, the ego was asked to indicate how often they communicated with each listed alter on social media platforms (e.g., Facebook) and socialized in other ways not related to game attendance. Respondents indicated social media communication and other socialization with alters not related to game attendance through a five-point Likert scale ranging from “Never” to “Daily.” The information gathered from this section allowed for the creation of three separate types of ties between the ego and alter—(1) game attendance, (2) social media interaction, and (3) other non-team-related interactions—to better determine the level of interactivity between the ego and alter.

The third section of the survey instrument (alter attributes) asked respondents to provide more information about each alter they identified, including the gender of the alter, whether the alter was a member in the same soccer supporters’ group as the ego, and the ego’s perception of the alter’s level of commitment to the specific soccer team. The perception of the alter’s commitment to the team was assessed on a five-point Likert scale ranging from “Extremely uncommitted” to “Extremely committed.” Alter attribute information allowed us to evaluate whether potential relationships between the egos and their alters occurred based on homophily, or shared attributes, such as gender, supporters’ group membership, and team commitment [8].

The fourth section of the survey instrument (alter–alter ties) asked respondents to assess whether the alters named by the ego in the first section shared relationships with one another (e.g., does Alter #1 share a relationship with Alter #2? Does Alter #2 share a relationship with Alter #3?). These relationships between alters focused on whether an ego’s alters attended games together and socialized together outside of team-related activities. This information allowed the complete construction of egocentric networks for each respondent, including the relationship between an ego and each of their alters and the relationships an ego’s alters have with one another. By including alter–alter ties, the full range of network statistics (i.e., density, structural holes) is available for examining each egocentric network.

The fifth section (team identification) and sixth section (soccer supporters’ group identification) of the survey instrument used items adapted from Mael and Ashforth’s [36] six-item organizational identification scale to assess a respondent’s level of identification with a team and supporters’ group. Scales used in sport research to measure team identification range from brief unidimensional scales to complex multidimensional scales [11]. Given that team identification and supporters’ group identification were just two variables out of many ego- and alter-level variables used to examine co-attendance, we opted to use Mael and Ashforth’s [36] scale due to its brevity, practical utility, and strong psychometric properties found within a wide variety of research contexts [37]. An example item from this scale adapted to measure team identification was, “When I talk about [insert team name], I usually say ‘we’ rather than ‘they.’” This same item when used to measure supporters’ group identification read, “When I talk about [insert name of supporter group], I usually say ‘we’ rather than ‘they.’” Each item in these two sections was measured using a five-point Likert scale ranging from “Strongly Disagree” to “Strongly Agree,” with higher scores indicating higher levels of team or supporters’ group identification. Information gathered from these responses helped determine the level of fandom exhibited by respondents and potential connections between identification levels and the number of alters identified by each respondent.

The seventh and final section of the survey instrument (demographic and behavioral information) asked respondents to address items such as number of games attended during the current and previous season, amount of time spent on social media related to the team, and tenure as a fan of the team and as a member of the supporters’ group. Respondents were also asked to indicate their age and gender. Information gathered in this section was used along with data collected from previous sections to determine potential relationships among variables such as the number of alters identified by respondents.

### 2.3. Data Analysis

Once the surveys were complete, the raw Qualtrics data were imported into E-Net [38]. Most general social network analysis software (e.g., UCINET) can calculate egocentric variables, but E-Net is designed specifically to examine egocentric networks. Using the guidelines presented by Halgin and Borgatti [39], three different matrices were created to properly situate and examine the egocentric data. First, an ego-level matrix that included a numerical identifier for each ego and ego-level variables was created. Second, an ego–alter tie matrix was created that contained each ego–alter tie, the associated attributes of that tie (i.e., tie strength), and the attributes of the alter. Each alter was presented in a separate row to ensure alter-level variables could be utilized in subsequent analysis. Finally, alter–alter ties were presented in the third matrix. With these three data files prepared for E-Net, we began examining the egocentric networks of soccer supporters.

As we had complete information on ego attributes, alter attributes, and alter–alter ties, we utilized the full potential of egocentric network analysis. According to Perry et al. [29], egocentric network analysis typically examines statistics related to ego–alter ties (i.e., network size, multiplexity, tie strength), alter attributes (i.e., heterogeneity, homophily), and alter–alter ties (i.e., density, structural holes). Table 1 reflects the egocentric network statistics calculated via E-Net.

With egocentric variables calculated, our data analysis shifted to formatting the data for multilevel modeling. Using HLM Version 7.03 [40], we followed the protocol established by Raudenbush, Bryk, Cheong, Congdon, and du Toit [41] for hierarchical linear modeling (HLM), a common technique for analyzing data with a nested structure. In multilevel egocentric networks, alters are nested in the ego [29,30,33]. HLM is similar to standard regression in that its purpose is to model a relationship between a dependent variable and a set of independent variables [32], yet HLM allows for within-cluster dependence. Each alter in a network represents a different Level-1 observation but undoubtedly shares some commonality with other Level-1 observations in the same cluster. In other words, when we examine a Level-1 outcome within egocentric networks, the dependent variable must be explained by variance at Level-1 while appreciating the dependence among units in the same cluster. Such dependence, which violates a core assumption of standard regression, is viewed not as a flaw in HLM but rather an opportunity to explore how both alter- and ego-level variables affect the outcome variables [42].

Perry et al. [29] outlined three conditions which must be satisfied to utilize multilevel modeling with egocentric networks: (1) the outcome variable must occur at the alter or ego–alter tie level of analysis; (2) ego-level observations must be independent of the other ego observations in the sample; and (3) there cannot be substantial overlap among the different egocentric networks. Our study meets these criteria, and thus we continued with preparing the data for HLM with a final dataset of 624 Level-1 observations (i.e., alters) nested in 119 Level-2 clusters (i.e., egos). The sample size in HLM differs from traditional regression sample size considerations. Maas and Hox [43] reviewed existing research on multilevel modeling sample sizes, showing a common number of Level-2 groups as around 50, with the Level-1 size of each group ranging from as high as 30 to fewer than 5. Echoing the conclusion of Browne and Draper [44], Maas and Hox [43] reported that a larger number of the Level-2 group (i.e., greater than 50) is more important than larger numbers of actors within each group. Based on these sample size expectations, we began modeling the 624 Level-1 actors nested in 119 Level-2 groups.

## 3. Results

We approached HLM modeling with a two-level model and consequently created two sub-models at Level-1 and Level-2. As a first step, we tested the level of dependence within the structured dataset. Using co-attendance as the dependent variable, we tested an unconditional model to provide estimates of the partitioning of variance at both Level-1 and Level-2 using full maximum likelihood estimation. The unconstrained model was significant (*X*^2^ = 674.95, *p* < 0.001); thus, we rejected the null hypothesis that all residuals are independent. Rejection of the unconditional model confirms the need for HLM to account for ego (Level-2)-induced dependencies. From the unconditional model, we then calculated the intraclass correlation (ICC), which provides a measure of the proportion of variability in co-attendance that exists between units [42], or the within-cluster correlation [45]. ICCs are calculated by dividing the between-group variance (*τ_00_*) by the sum of the between-group variance and within-group variance (*σ*^2^). The ICC was 0.4713, suggesting that over 47% of the variance in co-attendance occurs at the ego level, with the remainder occurring at the alter level.

Based on the unconditional model, we continued to the second model by including Level-1 variables. To test the relationship between alter-level variables and co-attendance, we created a random coefficient model to examine mean differences across alters within a particular ego. Using model fit deviance testing, we included Level-1 variables and tested whether they increased the fit of the model. Social media communication was entered first as a group-centered mean variable and significantly improved the fit of the model, based on a chi-square deviance test (*X*^2^ = 27.03, *p* < 0.001). Outside socialization was entered next as a group mean-centered variable and significantly improved the fit of the model (*X*^2^ = 10.27, *p* < 0.01). Alter commitment, centered on the group mean, was the third Level-1 variable and showed a significantly improved fit of the model (*X*^2^ = 139.123, *p* < 0.001). The same model fitting technique was used for same supporters’ group as an uncentered variable (*X*^2^ = 118.453, *p* < 0.001), alter degree centrality as a group-centered variable (*X*^2^ = 6.21, *p* < 0.05), and alter gender uncentered (*X*^2^ = 21.66, *p* < 0.001), which were all significant and included in the model. Finally, to test the Level-1 effect of relational classification, we included spouse, family, and co-worker in the model—not centered because they are binary variables. We included three relational classification categories and used friend as the reference variable for interpreting results from the other categorical variables. Including the relational classifications into the model did not significantly improve the model fit (*X*^2^ = 2.65, *p* > 0.50); therefore, the relationship variables were excluded from the model. The alter-level variables social degree centrality as a group-centered variable (*X*^2^ = 3.61, *p* = 0.064), gender homophily uncentered (*X*^2^ = 0.1423, *p* > 0.50), and alter structural holes as a group-centered variable (*X*^2^ = 0.05, *p* > 0.050) also did not improve the model fit and were thus excluded from the model.

The final Level-1 model was significant (*X*^2^ (92) = 786.68, *p* < 0.001), and a deviance test confirmed an improved fit over the unconditional model (χ^2^ (6) = 939.97, *p* < 0.001). Results for predictor variables are provided in Table 2. To calculate the effect size of the Level-1 model, we calculated the variance explained by including the alter-level variables; thus, we divided the difference between the null model within-group variance (*σ*^2^*_Null_*) and Level-1 model within-group variance (*σ*^2^*_Level1_*) by the null model within-group variance (*σ*^2^*_Null_*). Through our inclusion of social media communication, outside socialization, alter commitment, same supporter group, alter degree centrality, and alter gender, the model with alter-level predictors explained an additional 39.21% of the variance in co-attendance compared to the conditional model.

In the third model, we removed the Level-1 variables and included ego-level (Level-2) variables using chi-square deviance testing to understand which variables increased the model fit. Supporters’ group member, uncentered, was entered first, demonstrating an improved model fit over the unconditional model (*X*^2^ = 11.00, *p* < 0.001). Using a step-by-step model fit strategy, social media hours (*X*^2^ = 4.13, *p* < 0.05), team fan years (*X*^2^ = 5.72, *p* < 0.05), age (*X*^2^ = 4.55, *p* < 0.05), gender (*X*^2^ = 4.97, *p* < 0.05), fan density (*X*^2^ = 23.63, *p* < 0.001), social density (*X*^2^ = 7.94, *p* < 0.01), and same group heterogeneity (*X*^2^ = 5.30, *p* < 0.05) each significantly improved the fit of the model and remained in the final model. Other variables that did not significantly improve the fit of the model were team identification (*X*^2^ = 0.04, *p* > 0.50), ego network size (*X*^2^ = 0.22, *p* > 0.50), broker (*X*^2^ = 0.03, *p* > 0.50), structural holes (*X*^2^ = 1.15, *p* = 0.21), relationship heterogeneity (*X*^2^ = 0.37, *p* > 0.50), and gender heterogeneity (*X*^2^ = 0.02, *p* > 0.50). Each of those variables was removed from the model. All binary variables were included as uncentered variables; other variables were entered as grand mean-centered variables.

The final ego-level model was significant (χ^2^ (84) = 282.07, *p* < 0.001) and significantly improved the model fit compared to the unconditional model (χ^2^ (8) = 67.28, *p* < 0.001). To calculate the effect size of the Level-2 predictors, we calculated the variance explained by including the ego-level variables, explaining an additional 63.243% of the variance in co-attendance compared to the conditional model. Significant effects for ego-level variables include supporters’ group member (*γ_01_* = 2.29, *se* = 1.03, *p* < 0.05), age (*γ_04_* = 0.11, *se* = 0.04, *p* < 0.01), fan density (*γ_06_* = 0.24, *se* = 0.04, *p* < 0.01), social density (*γ_07_* = −0.11, *se* = 0.04, *p* < 0.01), and same club heterogeneity (*γ_08_* = 6.72, *se* = 3.07, *p* < 0.05). Gender was approaching statistical significance as a Level-2 variable (*γ_05_* = 1.78, *se* = 1.03, *p* = 0.08). Social media hours (*γ_02_* = 0.17, *se* = 0.17, *p* = 0.32) and team fan years (*γ_03_* = 0.63, *se* = 0.47, *p* = 0.18) were not statistically significant in Model 3.

In our fourth and final model, we combined ego- and alter-level variables. All alter-level variables were group-centered, and all ego-level variables were grand mean-centered, except for binary variables, which were not centered. The final model was significant (χ^2^ (84) = 417.11, *p* < 0.001) and represented a significantly better fit than the unconditional model (χ^2^ (14) = 372.84, *p* < 0.001). In the combined model, supporters’ group member (*γ_01_* = 1.32, *se* = 1.00, *p* = 0.18) and same club heterogeneity (*γ_08_* = 2.16, *se* = 2.83, *p* = 0.16) became non-significant at the ego level when compared to Model 1. Moreover, social media (*γ_10_* = 0.35, *se* = 0.19, *p* = 0.07) and alter gender (*γ_50_* = −0.90, *se* = 0.51, *p* = 0.07) approached statistical significance at the alter level in the combined model. All other variables remained consistent with previous models. Full results for the combined model are provided in Table 2.

## 4. Discussion

### 4.1. Theoretical Implications

Previous studies have investigated the relationship between fans and teams through the team identification conceptual framework [11,12]. Yet, researchers have found fan-to-fan relationships also play an important role [13,14,16] and, in some instances, are more important than team identification in explaining attendance behavior [15]. This research has frequently investigated social networks to understand the relationships formed between and among fans—how they combine to form networks as well as how these networks and shared relationships influence behaviors [13].

Co-attendance served as the primary dependent variable in our study, which centered on fan-to-fan relationships, rather than dyadic connections between fans and the team or brand. Lock and Funk [28] proposed the multiple in-group identity framework to describe the hierarchical identities surrounding teams, fan groups, and relational groups formed by fans with their friends, family members, co-workers, and other team supporters. We sought to better understand how interactions and personal characteristics of soccer club supporters influenced their attending games together. This required us to assess alter and ego variables individually as well as together to form a more complete picture of these networks.

We built upon the work of Katz et al. [34], who used multilevel modeling to understand co-consumption in the form of emotional support among fans, finding that supporting the same team, communicating in person, and sharing relationships with friends corresponded positively with emotional support. Our study also explored the effects of interpersonal interactions and shared personal characteristics between egos and their alters on co-consumption, in this case co-attendance. We examined four models, and the combined model of alter- and ego-level characteristics revealed several variables of significance. These models indicated co-attendance, or egos attending games with their alters, has variance at both the alter and ego levels of analysis. The model focused on alter-level characteristics explained over a third of the available variance in co-attendance. The model centered on ego-level characteristics accounted for almost two thirds of the potential variance. The combined model of ego- and alter-level characteristics resulted in six salient variables for consideration. These findings highlight the importance of analyzing both alter and ego variables to understand sport consumption behavior.

Salient alter-level characteristics included the perceived level of an alter’s commitment to the team, shared membership in a supporters’ group, and number of connections shared by alters with other alters in the same egocentric network. Perceptions of an alter’s level of commitment to the team were the most noteworthy. When an ego viewed a particular alter as having higher levels of commitment than other alters in the network, the ego attended more games with the highly committed alter. While alter commitment proved significant, ego team identification did not. This finding aligns with Yoshida et al. [15], who also reported team identification as a non-significant variable in their study of soccer fans. For understanding co-attendance, the commitment levels expressed by alters are more salient than those personally experienced by the ego.

In addition to alter commitment, the social nature of alters is important, as evidenced by the significance of the same supporters’ group and alter degree centrality. When an ego shares supporters’ group membership with an alter and this alter knows and connects with other alters, the ego attends more games with this alter than alters who do not share supporters’ group membership and know less of the other alters. Yet, moving from the ego-level model to the combined model showed the ego’s personal membership in the supporters’ group is no longer significant, perhaps “cancelled” by the importance of sharing membership in the same supporters’ group with alters. Katz et al. [14] noted the importance of fan-to-fan relationships to understand attendance. Likewise, Yoshida et al. [15] found the importance of fan relationships, rather than team identification, in predicting game attendance. This also follows the multiple in-group identify framework [28] and the presence of subgroups within a superordinate group. This framework suggests individuals can be members of some groups but not others. For example, fans could support a team and attend games with family and friends but not participate in a supporters’ group. Thus, the value of connections formed with and among alters in the ego’s network may supersede both identification with the team and individual group membership.

We found that when an alter has team-related relationships with other alters, the connected alter attends more games with the ego. The density of the outside socialization network, conversely, had a negative relationship with co-attendance. As alters socialize with alters outside of the team context, they attend fewer games with the ego. Significant ego characteristics also emerged, including age, fan network density, and outside socialization density. First, the older the ego, the stronger their co-consumption ties with alters. Second, the denser the fan network, the stronger the co-attendance. Density is a measure of cohesion, meaning it assesses the degree to which alters have ties with other alters in an egocentric network. The findings suggest maybe it is not just about social ties between alters, but rather about the role of the team in connecting the alters. This aligns with Lock and Funk [28], who argued the team serves as the superordinate group, allowing for subgroups and relational subgroups to occur within that superordinate identity. At the alter level, creating an environment with other fans who are committed to the team, belong to the same supporters’ group, and share connections with others in the network correlates with higher co-attendance. Ego-level characteristics can create the same effect, where older fans who have more connections to others who attend games and interact outside of game attendance also attend games with others in their network more frequently.

This multilevel investigation moves away from previous sport research (and marketing research more generally) focused on individual consumption behaviors, particularly those occurring between the consumer and a brand, where the individual and their actions are the sole focus. Instead, we examined these behaviors of individuals in conjunction with the behaviors of those with whom they interact through co-attendance at soccer games. Prior fan interaction research [9,13,15,16,31] highlighted the importance of fan-to-fan communities and how the role of relationships with others can influence sport consumption behaviors. Our study also illustrates the importance of fan communities on sport consumption, with the potential for fans to form groups consisting of supporters’ group members and others with whom they interact. Positive connections formed with group members correspond with increased game co-attendance.

Non-significant findings also emerged in our analysis. These findings involved egocentric network variables such as social degree centrality; gender homophily; alter structural holes; ego network size; and heterogeneity of relationship, gender, and club membership. Social degree centrality quantifies how embedded an individual is within a network. In this study, social degree centrality corresponded to the number of people attending games with an individual. Homophily indicates the similarity between two individuals within a network, such as two people of the same gender attending games together. Structural holes result from the absence of ties among network members. In this research context, that could indicate alters attending games with the ego but not with one another. Ego network size quantifies the numbers of alters with whom an ego is connected—e.g., the number of individuals attending games together. Heterogeneity indicates the variability of network members based on attributes such as relationship classification, gender, and supporters’ group membership. In this study, non-significance indicated a limited range of variability on these factors among individuals within the egocentric networks.

Our study extends the work of Katz et al. [34], who also used social network analysis and HLM to examine similar network variables. They reported non-significant findings for network size, brokerage, and heterogeneity in their model combining ego- and alter-level variables. Researchers should continue to explore these network-specific variables to determine whether certain sport, geographic, and fandom contexts generate unique outcomes. The continued emergence of non-significant network findings in future sport studies might suggest certain egocentric network characteristics, such as centrality, homophily, structural holes, network size, and heterogeneity, have limited effects on specific fan behaviors. Future studies can assist researchers in developing egocentric network theoretical frameworks across a variety of sport contexts.

### 4.2. Methodological Implications

Next, we discuss the methodological implications as they relate specifically to the use of egocentric social network analysis. “Sport consumption is not merely an individual activity; rather, individual sport consumption needs to be conceptualized as part of the larger network of consumers within which individuals belong” [31] (p. 217). Few people attend games alone [31]. Thus, it is important to analyze fan-to-fan interactions in more detail. Our study examined co-attendance occurring with soccer supporters’ group members and other fans. Previous research has examined soccer supporters’ groups [4,5,6,7], but few studies have used social network analysis to explore the shared relationships occurring within these groups and, more specifically, how this correlates with sport consumption behaviors [34]. The current study represents one of the first to incorporate and conduct egocentric network analysis in this context.

We adopted this approach by focusing on fans, their relationships with other fans within and outside supporters’ groups, and how this translated into game co-attendance. This focus aligns with social network analysis, which centers on interactions among individuals, groups, and organizations rather than the individual characteristics and actions of these network members [29]. Egocentric network analysis represents an appropriate method to conceptualize and operationalize these group connections and investigate their potential effect on sport consumption behaviors. Crossley et al. [30] argued three advantages of egocentric network analysis, including the ability to understand intersecting and overlapping social circles, which mirrors the multilevel framework proposed by Lock and Funk [28]. Adopting an egocentric approach that includes different subgroups and relational groups as well as alter data is important to capture the full network picture [29]. Simply asking egos to report the number of games they attended with other fans without examining these groups, related alter characteristics, and relationships shared with other alters would have caused us to overlook a significant part of the story [34].

We argue the importance of utilizing egocentric networks, particularly within this supporters’ group setting. Analysis of sociocentric networks requires establishing a network boundary from the outset [29]. We did not know in advance the complete roster of members within the supporters’ groups nor the potential interactions they had with non-members. Having the boundaries emerge through study participant responses allowed us to create inclusive networks [29,30]. As such, our sample includes both members of supporters’ groups and non-members. Focusing solely on a set group of members (e.g., official members of a supporters’ group) would have caused us to overlook other relationships formed between egos and alters as well as between alters and alters who might not be members of the same group yet played an influential role in game attendance. Positioning supporters’ groups within this framework and using egocentric network analysis to understand fans and their relationships with the team and other fans present an exciting combination of theoretical and methodological perspectives.

### 4.3. Practical Implications

From a practical perspective, this study represents one of the first to examine the effects of soccer supporters’ group membership on co-attendance. The findings provide insights for leaders of professional soccer clubs in the United States about the potential benefits of supporters’ groups to their respective organizations. These soccer clubs seek to generate revenues, and game attendance represents an opportunity to do so. The findings of this study suggest organizational leaders would benefit from encouraging fans to interact together around the superordinate identity of the team. Thus, an opportunity exists for team leadership to focus on specific variables key to co-attendance, namely, from this study: alter commitment, alter degree centrality, same supporter group membership, and fan density. Essentially, soccer club leaders should work to encourage more fans, particularly those who have higher levels of commitment and share ties with others from the same supporters’ groups, to attend games together.

Finding ways to increase co-attendance could have a positive effect on revenues as more fans of the same soccer club collectively engage in this behavior. Using information from this study, soccer club leaders could meet with supporters’ group members to learn more about their sentiments regarding the team and attendance. They could explore reasons for why supporters’ group members join these groups as well as why they attend games and with whom. Soccer club leaders additionally could probe for ways to increase this attendance. The study’s introduction noted some aspects of attendance germane to soccer supporters’ groups such as sitting together in specified parts of the stadium, carrying flags and banners, participating in various cheers and chants, and playing music. These activities rely upon supporters’ group members acting collectively. Learning more about these and other attendance rituals, as well as ways to support these efforts, could aid in increasing co-attendance as members engage in these behaviors together. Organizational activities facilitating these efforts could include officially setting aside designated seating for supporters’ group members as well as offering promotions and group ticket pricing. Identifying these and other ways to support and promote these efforts could benefit both the supporters’ groups and the soccer clubs they support.

### 4.4. Limitations and Future Research

This study had several limitations. First, this study utilized a voluntary response sample [46], meaning not every member of the target population had a nonzero probability of participation. This sampling technique is popular when it is not reasonably possible to gain access to an entire target population [46]. This was the case within this research context, where it was not possible to access the entire population of supporters for each soccer team included in this study. However, there are certain disadvantages to this type of sampling procedure, most notably the presence of coverage error [47]. Therefore, the sample included in this study may have over- or under-represented certain segments of the target population, thereby limiting the generalizability of the results to the target population group. Another risk from voluntary response sampling is the possibility of abnormally high response rates from participants who hold strong opinions on a particular subject [47]. However, prior research [48] noted this risk is minimal when the topic examined is not of a controversial nature. There is no indication in the past literature that co-attendance or association with a soccer supporters’ group for a team based in the United States represents a controversial topic.

Additional limitations include surveying fans only once at the start of the season. Their shared relationships and other alter and ego characteristics could have changed at different points during, before, or after the season. We also collected data from individuals associated with soccer supporters’ groups for two professional soccer teams located in the same region of the United States. Fans of other teams and supporters’ groups could have provided varying responses. Additionally, we focused on professional soccer, a sport with relatively new popularity in the United States compared to professional baseball, basketball, football, and hockey. Collecting data from fans of other sports might have yielded different results.

Future research could address these limitations and add to the burgeoning literature using egocentric network analysis to investigate sport settings. First, studies could adopt a longitudinal approach. Lock and Funk [28] noted the nature of relationships and groups can change over time, and Yoshida et al. [15] surveyed sport fans twice during a soccer season. Collecting data at multiple points could unearth insights into how these groups evolve. An opportunity also exists to incorporate more supporters’ groups. Future research could conduct a broader analysis of supporters’ groups from a variety of teams to identify potential similarities and differences among them. Researchers could explore fans of teams with longer tenures in other sports to document how these groups compare to those found within North American professional soccer. Studies could also incorporate findings from supporters’ groups for professional soccer teams in Europe, which have longer histories and traditions compared to those examined in this study.

In the current study, we examined several relationship types (e.g., in-game attendance, social media usage) and relational categories (e.g., friend, co-worker). We did not find significance with all relationship types and categories; however, we believe an opportunity exists to continue this exploration. Katz et al. [34], in their multilevel analysis of egos and alters, looked more specifically at variables such as communicating in person, via text, and through social media. They also examined relationships between family members, friends, co-workers, and strangers. Future research could include additional variables to provide a more nuanced view of the activities occurring within networks and among network members. Studies could also include other behaviors such as hosting watch parties and traveling to away games. Finally, our study and previous ones [14,15,34] focused on the positive aspects of fan interactions such as attending games and providing emotional support. However, negative attitudes and behaviors toward competing supporters’ groups and fans could emerge. Future research could investigate the potential for in- versus out-group sentiments and exclusionary behaviors between these groups.

## 5. Conclusions

Professional soccer has grown exponentially in the United States. Soccer supporters’ groups have correlated with franchise expansion and contributed to sizeable increases in game attendance. The current study examined soccer supporters’ groups for two teams, using egocentric network analysis and multilevel modeling. With this approach, we explored the characteristics of network egos and their alters and how these combined to influence game co-attendance. The findings revealed positive effects of perceived alter commitment, shared supporters’ group membership, age, and alter–alter interactions on co-attendance. The results provide both theoretical and methodological implications by highlighting the importance of fan-to-fan communities to teams and their game attendance as well as answering the continued call to use egocentric network analysis in sport fan contexts.

## Figures and Tables

**Table 1:** Egocentric network variables [29].

Variable	Definition	Example
Ego–Alter Ties		
Network Size	The number of alters to whom a given ego is connected	Co-attendance network size
Multiplexity	Multidimensionality of interpersonal ties	Socialization outside of team-related activities
Tie Strength	Intensity of tie or frequency of interaction	Social media communication surrounding superordinate identity (i.e., team)
Alter Attributes		
Composition	Count of alters in a network based on a category or role	Gender composition of ego network
Homophily	Similarity between ego and alter on specified attribute	Member of same supporters’ group
Heterogeneity	Similarity of alter to alter on a specific attribute	Relational heterogeneity among family, friends, and co-workers
Alter–Alter Ties		
Density	The degree of connectedness among alters	Social tie density
Structural Holes	Absence of ties between alters in an ego’s network	Alter structural holes

**Table 2:** Hierarchical linear modeling results for co-attendance.

Variable	Model 1:		Model 2:		Model 3:		Model 4:	
	Unconditional Model		Alter-Level Characteristics		Ego-Level Characteristics		Combined Model	
	M	SE	M	SE	M	SE	M	SE
Level 1								
Intercept (*γ_00_*)	9.58 ***	0.71	7.32 ***	0.63	7.40 ***	0.90	6.34 ***	0.85
Social Media (*γ_10_*)			0.31	0.20			0.35 ^†^	0.19
Outside Socialization (*γ_20_*)			0.37	0.28			0.19	0.23
Alter Commitment (*γ_30_*)			2.20 ***	0.28			2.31***	0.28
Same Supporter Group (*γ_40_*)			6.48 ***	0.97			5.79***	0.90
Alter Degree Centrality (*γ_50_*)			0.44 *	0.20			0.49 *	0.19
Alter Gender (*γ_60_*)			−1.0	0.53			−0.90 ^†^	0.51
Level 2								
Supporter Group Member (*γ_01_*)					2.29 *	1.03	1.32	1.00
Social Media Hours (*γ_02_*)					0.17	0.17	0.19	0.14
Team Fan Years (*γ_03_*)					0.69	0.47	0.64	0.43
Age (*γ_04_*)					0.11 **	0.04	0.11 **	0.04
Gender (*γ_05_*)					1.78 ^†^	1.03	1.71 ^†^	0.99
Fan Density (*γ_06_*)					0.24 ***	0.04	0.19 ***	0.04
Social Density (*γ_07_*)					−0.11 **	0.04	−0.07 *	0.04
Same Club Heterogeneity (*γ_08_*)					6.72 *	3.07	2.16	2.83
Variance								
Within-Group (*σ*^2^)	43.16		26.24		43.26		26.21	
Between-Group								
Intercept (*τ_00_*)	38.38		29.13		14.14		14.72	
Intraclass Correlation	0.4713							
*r* ^2^			0.3921		0.6324			
Model Deviance (df)	3935.55 (3)		3614.15 (9)		3868.26 (11)		3562.84 (17)	
Base Model Comparison			χ^2^(6) = 939.97 ***		χ^2^(8) = 67.28 ***		χ^2^(14) = 372.84 ***	

*** = *p* < 0.001. ** = *p* < 0.01, * = *p* < 0.05, † = *p* < 0.10.

## Data Availability

The data presented in this study are available on request from the corresponding author.
